# Gendered dynamics in outpatient psychotherapy: An interpretative phenomenological analysis of female patients' and male therapists' experiences

**DOI:** 10.1111/papt.70005

**Published:** 2025-08-12

**Authors:** Erwin Schweitzer, Yvonne Schaffler, Thomas Probst, Elke Humer, Christoph Pieh, Brigitte Schigl

**Affiliations:** ^1^ Department for Psychosomatic Medicine and Psychotherapy University for Continuing Education Krems an der Donau Austria; ^2^ Division of Psychotherapy, Department of Psychology Paris Lodron University Salzburg Salzburg Austria; ^3^ Department Psychology and Psychodynamics Karl Landsteiner University of Health Sciences Krems an der Donau Austria

**Keywords:** female patients, gender, interpretative phenomenological analysis, male psychotherapists, relational phenomenology

## Abstract

**Objectives:**

Gender dynamics within the patient–therapist relationship can influence the therapeutic process in meaningful ways. However, the female patient–male therapist dyad has received limited attention in recent psychotherapy research. This article explores how female patients and their male therapists experience the role of gender in outpatient psychotherapy.

**Design:**

This study is a qualitative subproject within a larger research initiative involving psychotherapists in private practice and their patients in Austria. Interviews with patients and therapists were conducted using a comprehensive, semi‐structured interview guide.

**Method:**

Interviews were conducted with ten participants from female patient–male therapist dyads. The data were analyzed using dyadic interpretative phenomenological analysis and interpreted through the theoretical lens of relational phenomenology.

**Results:**

The analysis identified four themes that illustrate participants' recognition of gender as an important factor influencing psychotherapy: (1) gender shapes the topics and interventions in psychotherapy, (2) the male therapist's gender as valuable for the female patient, (3) the female patient's gender as valuable for the male therapist and (4) attraction between male therapist and female patient. A contrasting fifth theme reflects perspectives suggesting that (5) gender plays a lesser role in psychotherapy.

**Conclusion:**

Participants offered varied and sometimes conflicting views on gender's role in outpatient psychotherapy. While they acknowledged its influence, some felt other factors held greater significance. These differing perspectives reflect diverse experiences of gender, which, if not adequately addressed in therapy, may hinder the therapeutic process.

## INTRODUCTION

The female patient–male psychotherapist dynamic likely played a pivotal role in the emergence of modern psychotherapy during the late 19th and early 20th centuries. This dynamic is exemplified by the well‐known case of Bertha Pappenheim (better known by her pseudonym, Anna O.) and her therapist, Josef Breuer, a mentor to Sigmund Freud. Pappenheim's case became central to ‘Studies on Hysteria’ (Breuer & Freud, 1895), co‐authored by Breuer and Freud. During her treatment, Pappenheim is credited with coining the term ‘talking cure’, a concept that significantly influenced the development of psychoanalysis (Brownstein, 2024).

While this specific patient–therapist pair holds a prominent place in the history of psychotherapy, the ‘most traditional gender pairing’ (Proctor, 2008, p. 88) of female patients and male therapists has received little recent attention from psychotherapy researchers. This lack of focus is particularly regrettable, as research suggests that the female patient–male therapist dyad may impact the therapeutic process, relationships, outcomes and associated risks and potentials (Schigl, 2018). In this study, we address this gap by exploring how both female patients and their male therapists experience the significance of gender within the psychotherapy process and relationship.

### Psychotherapy research on female patient–male therapist dyads

No recent research has focused exclusively on the female patient–male therapist dyad in psychotherapy, to the best of our knowledge. However, several studies have examined the role of patient and therapist gender, as well as gender pairings, in relation to patient preferences, satisfaction, therapeutic outcomes, the therapeutic relationship and the therapy process.

Patients' preferences for the gender of their therapists have been shown to be gendered, according to several studies. This preference is particularly pronounced among female patients, who tend to prefer female therapists across numerous studies (Furnham & Swami, 2008; Ilagan & Heatherington, 2021; Landes et al., 2013). Schweitzer et al. (2024) found that female patients' preference for female therapists stems from perceiving them as possessing gendered resources relevant to therapy. These resources include presumed shared life experiences, as well as traits and skills traditionally associated with femininity, such as being more empathic and caring.

Research on patients' satisfaction and therapy outcomes appears to be inconsistent. Some quantitative studies have found that patients were less satisfied in gender‐heterogeneous pairings (Johnson & Caldwell, 2011; Schigl, 2014). Similarly, a study by Leitner et al. (2013) found that female patients paired with male therapists were less satisfied and at a higher risk of harmful side effects of therapy. These patients reported greater stress related to therapy and higher dropout rates compared to other gender pairings. In line with these findings, a recent cross‐sectional study (Schmalbach et al., 2022) concluded that both female and male patients experienced less improvement in their quality of life when treated by male therapists. The authors suggested that this may be due to female therapists demonstrating greater responsiveness and empathy towards their patients. However, contrasting evidence comes from the majority of psychotherapy research, which has shown that the therapist's gender or the gender match between patient and therapist does not significantly impact therapeutic outcomes (Blow et al., 2008; Lambert, 2016; von Wyl et al., 2021; Zlotnick et al., 1998).

Other studies have examined differences in therapy styles between male and female therapists and the impact of these differences on therapy outcomes. According to patients and external raters, male therapists tended to adopt a more confrontational, direct and problem‐focused approach (Gehart & Lyle, 2001; von Wyl et al., 2021). In contrast, female therapists were perceived as more supportive, caring and emotion‐oriented. A randomized controlled trial linked gendered therapy styles to outcomes, finding that female patients showed greater improvement in supportive therapies, while male patients benefited more from confrontational therapies (Ogrodniczuk & Staats, 2002). This suggests that gendered therapy styles, as practiced differently by male and female therapists, can influence psychotherapy outcomes. However, somewhat contradicting these findings, Owen et al. (2009) argued that some therapists, regardless of their gender, appeared to be more effective at treating male patients, while others were better suited to treating female patients.

Several studies have explored differences in the therapeutic relationship between the female patient–male therapist pairing and other gender combinations. Proctor (2008) highlighted that this dyad is particularly prone to reproducing societal power inequalities between women and men, driven by dynamics of hegemonic masculinity and gendered stereotypes. Female patients may be more inclined to adopt obedient or deferential behaviours due to the male therapist's perceived social authority, whereas male therapists may need to make significant efforts to foster a more equitable therapeutic relationship and to be seen as caring and empathic. In Schigl's (2019) study, male therapists noted that building a therapeutic relationship was generally easier with female patients than male patients. They observed that female patients tended to be more talkative and appreciative towards them. However, male therapists in this pairing also reported more frequent experiences of erotic dynamics with their female patients compared to other gender combinations. Additionally, male therapists often attempted to act as a male role model for their female patients due to the patients' relationship problems. Compared to gender–homogeneous pairings, addressing certain topics—such as sexuality, gender identity, reproduction, corporeality, intimate relationships, division of care and domestic work within the family—was described as more challenging. Similarly, Tolle and Stratkötter (1998, p. 259) found that therapists in gender–heterogeneous pairings experienced the therapeutic relationship as more distant, alien and cautious. They also reported that it required greater effort to empathize with their patients compared to working in gender–homogeneous dyads.

Recent research relevant to the female patient–male therapist dyad has found that female patients tend to prefer female therapists and are often less satisfied with therapy when paired with male therapists. Regarding therapy outcomes, findings have been inconsistent, although differently gendered therapy styles may influence outcomes. The therapeutic relationship within the female patient–male therapist dyad appears to be shaped by a range of factors, including gender inequalities and stereotypes, feelings of otherness, difficulties in addressing intimate topics, experiences of sexual attraction, ease of relationship building and the therapist's desire to serve as a positive male role model. Notably, we found no studies that explore the experience of gender in the therapeutic relationship from both the patient's and therapist's perspectives in a dyadic analysis. Given the increasing recognition among psychotherapy researchers that therapy is fundamentally an interpersonal experience shaped by dynamic, dyadic interactions (Atzil‐Slonim & Tschacher, 2020), it is crucial to conduct studies that explore this aspect of the therapeutic process and relationship.

### A relational phenomenology of psychotherapy

We adopt a phenomenological perspective to analyse our research phenomenon, aiming to uncover how gender is experienced—beyond mere description—to reveal its deeper structures of meaning and relationality. By examining gendered experiences within the therapeutic process, we gain insight into how cultural practices and norms shape therapeutic relationships. Recognizing the diversity within phenomenology (Schnegg, 2023), our approach integrates relational, embodied and ecological perspectives (Fuchs, 2019; Ingold, 2017; McNay, 2004; Petzold, 2003; Zigon, 2023), which view individuals as embodied, responsive subjects interconnected with others and embedded in socio‐ecological and historical contexts. For simplicity, we refer to these related approaches as ‘relational phenomenology’.

In the context of psychotherapy, German philosopher and psychiatrist Thomas Fuchs (2019) has expanded upon three key theoretical concepts: the phenomenal field, body memory and lived space, which provide a robust foundation for our approach. The *phenomenal field* encompasses everything we subjectively experience through our lived body in the present moment. It is shaped by our interactions with other beings and things in our immediate environment, which engage with our perceptions, movements, emotions, responses and bodily resonances. This field is further informed by past experiences embedded in our *body memory*. Body memory refers to the embodied patterns of perception, movement, response, thought, emotion and longing that develop through an individual's life experiences. It also includes the relational skills used in direct interactions with others, inscribed in the intercorporeal memory. These embodied patterns and relational skills shape our interactions and influence how we perceive and respond to therapeutic encounters. Lastly, the *lived space* refers to the unique environment—both human and more‐than‐human—that a person inhabits. It is shaped by cultural practices and ecological affordances, which outline the collective possibilities and constraints that are inherent in any given environment.

Although Fuchs' phenomenological framework allows for the influence of cultural and societal factors (Fuchs, 2012), it does not explicitly address gender issues. To bridge this gap, we extend Fuchs' concepts by incorporating a relational phenomenology of gender, drawing on the work of French sociologist Pierre Bourdieu (2002). Gendered cultural norms and practices influence the phenomenal field, intertwining with past experiences within the lived space and becoming inscribed in the body's memory (McNay, 2004; Schweitzer et al., 2024). Within this theoretical framework, gender is a ‘lived relation’ (McNay, 2004, p. 185), shaped by subjective experience, intersubjective relations and societal structures, while remaining entangled with—but not determined by—the materiality of the body (Schigl, 2018).

From a relational phenomenological perspective, psychotherapy alleviates suffering by engaging patients and therapists in an ‘interactive phenomenal field’ (Fuchs, 2019, p. 5) where they correspond not just as ‘talking heads’ but through their entire lived bodies. Within this dynamic, the therapist–patient dyad forms a ‘momentary knotting together of relational forces’ (Zigon, 2023, p. 128). In this knotting, patterns of relating rooted in body memory are reactivated and may be transformed through new experiences (Petzold, 2003). As all human experiences this process is not gender‐neutral but deeply gendered (Schigl, 2018; Schweitzer et al., 2024). Through therapy, the patient's lived space may evolve, enabling greater autonomy, expanded possibilities, and the capacity for healthier, more fulfilling relationships (Fuchs, 2019).

## MATERIALS AND METHODS

### Design

This study is a qualitative subproject of the larger ‘Process and Outcome of Psychotherapy in Private Practice’ (POPP) project (Schaffler et al., 2024), launched 2020 and conducted across various federal states in Austria. A comprehensive interview guide was used, covering topics such as access to psychotherapy, therapy expectations and goals, key moments in both helpful and challenging phases, changes experienced during therapy and the role of gender in the therapeutic relationship and process. This article focuses on the latter, the significance of gender. Specifically, this section of the interview guide explored (1) participants' perceptions of their therapist's or patient's gender, (2) their experiences of therapy with someone of a different gender and (3) situations where gender significantly influenced the psychotherapy process.

The semi‐structured interviews were conducted by psychotherapists‐in‐training enrolled in psychotherapy study programs, who received specialized training as interviewers. Between the summer of 2021 and autumn of 2023, these interviewers carried out face‐to‐face interviews at locations chosen by the participants, including psychotherapy practices, private homes and public spaces. This article focuses on the qualitative phenomenological analysis of a subsample of ten semi‐structured interviews with five patients and five therapists, representing all female patient–male therapist dyads from the sample. The interviews, lasting between 38 and 110 min, were recorded and transcribed verbatim by the interviewers. Participants provided written informed consent, and the study was approved by the Ethics Committee of the University of Continuing Education Krems (UWK), Austria (EK GZ 28/2018‐2021).

### Participants

The participants in this study were licensed psychotherapists and their legally adult patients in private practice. Psychotherapists were invited to participate through information shared via email, newsletters, social media and online workshops. Following this, the participating therapists recruited patients by inviting the next patient starting therapy to join the study, with no preselection based on diagnosis. Each patient was interviewed once, randomly selected from either the initial, mid‐point or concluding stages of their therapy. After conducting the patient interviews, the interviewers sought the patient's consent to interview their therapist. Following this, the interviewers proceeded to interview the patients' therapists.

The analysed sample included five patients who self‐identified as female (Table 1). In their therapists' interview statements, these patients were also ascribed a female gender identity. Conversely, the five therapists self‐identified as male and were ascribed a male gender identity by their patients, as inferred from the patients' interview statements (Table 2). The patients, aged between 20 and 59 years, resided across four Austrian federal states. Their educational backgrounds varied: one held a high school diploma, another completed vocational training, and three had university degrees. Each patient attended between 6 and 20 psychotherapy sessions with their respective therapist. This range represents the initial phase of psychotherapy, a period during which gender may exert a more pronounced influence (Schigl, 2018). This contextual factor is important to consider when interpreting the study's findings. Among the therapists, two practiced a psychodynamic approach, while three followed a humanistic orientation. Each therapist treated one patient from the sample.

**TABLE 1:** Patient sample characteristics.

Category	Description	Number of patients
Therapy sessions	6–10 sessions	2
	11–15 sessions	2
	16–20 sessions	1
Diagnosis (multiple entries possible)	Depression	4
	Anxiety	5
	Personality disorder	1
Age (years)	21–30	2
	31–40	1
	41–50	1
	51–60	1

**TABLE 2:** Therapist sample characteristics.

Category	Description	Number of therapists
Work experience (years)	<5	1
	10–20	2
	≥25	2
Therapeutic orientation	Psychodynamic	2
	Humanistic	3
Age (years)	41–50	1
	51–60	2
	61–70	2

### Analysis

The interview transcripts were analysed using the qualitative data analysis software ATLAS.ti Scientific Software Development GmbH (2024). The methodological approach applied was interpretative phenomenological analysis (IPA), which focuses on ‘the detailed examination of human lived experience’ within both personal and social contexts (Smith et al., 2022, p. 26). In line with the relational phenomenological approach outlined earlier, this study draws on recent developments in multi‐perspectival IPA (Larkin et al., 2019). This variant of IPA extends traditional IPA by incorporating systemic and relational perspectives, emphasizing that ‘important meanings are often located “in between” persons’ (Larkin et al., 2019, p. 192).

This study follows the steps of IPA as outlined by Smith et al. (2022), with adaptations for multi‐perspectival designs suggested by Larkin et al. (2019) and Radley et al. (2023). While the first author initially conducted the analysis, the final step involved collaboration with the second author. (1) The initial step involves becoming familiar with the data by listening to the audio recordings of the interviews and reading the corresponding transcripts. The researcher then made memos attached to relevant text passages related to the research question. (2) In the second phase, the researcher identified Experiential Statements from the participants that related to their experiences of gender in therapy and labelled them. (3) The next stage involves searching for connections between the Experiential Statements. Related statements are grouped together and named, forming Personal Experiential Themes for each research participant. The first author began these first three phases with a patient interview and then replicated the steps for the corresponding therapist interview within the same dyad. (4) Once Personal Experiential Themes were developed for both the patient and the therapist, the analysis proceeded to the dyadic level to identify Dyad Experiential Themes. In this phase, the researcher examined relationships between the Personal Experiential Themes of the same dyad. Related themes were clustered and named, focusing on shared experiences rather than mere similarities (Radley et al., 2023) to develop Dyad Experiential Themes. Steps 1 to 4 were repeated in the same sequence for all participants and dyads. (5) The researcher then looked for relationships between all the dyads. Dyad Experiential Themes that had connections were grouped together, and Group Experiential Themes were developed by exploring both the similarities and differences in perspectives regarding shared experiences. (6) Although the first author initially guided the analytical process, the analysis was carried out collaboratively by a male–female researcher dyad. To ensure the interpretations reflected the experiential data, the second author thoughtfully reviewed the themes developed by the first author in relation to the data. Any differences were resolved through open, reflective discussions and repeated engagement with the transcripts, leading to a shared understanding and refinements to the analysis as needed.

Larkin et al. (2019) highlight that multi‐perspectival analysis raises unique ethical considerations related to internal anonymity. Linking statements from members of the same dyad may lead participants to recognize one another, and even when pseudonyms are used, participants might attempt to cross‐reference the text based on these identifiers. This is particularly concerning in psychotherapy research, where such recognition could interfere with the delicate dynamics of the patient–therapist relationship. To address this, we adopted an approach inspired by Loaring et al. (2015) and chose not to use pseudonyms in presenting our results. In contrast to Haskayne et al. (2014), we connect perspectives within a dyad under specific themes, as we believe that this linkage offers a deeper insight into the phenomenon we are exploring. While this approach prevents readers from identifying dyads across the entire article, we prioritize the protection of our participants. Additionally, the interviews were translated from Austrian German into English, further minimizing the likelihood of participants identifying each other.

### Researchers and reflexivity

The analysis was carried out by a collaborative team consisting of the first and second authors, both of whom were involved in the interview process and had developed a strong connection to the research field. The first author identifies as male, and the second author as female. As psychotherapy researchers, integrative psychotherapists and social anthropologists, they bring a diverse set of experiences and perspectives to the project. As integrative therapists, they approach the work without a bias towards any single psychotherapy orientation, while their anthropological background leads them to value the importance of context and individual differences. This approach is woven into the theoretical and methodological framework of the study.

From a prior study (Schweitzer et al., 2024), they had observed that female patients often felt more comfortable with female therapists, perceiving their skills and experiences as ‘feminine’. This led to a genuine curiosity in this study about whether women working with male therapists might find certain ‘masculine’ qualities in their therapists that they valued. Building on the literature, they also suspected that the erotic dynamic could be relevant in such a dyad. These assumptions were partially confirmed; both authors were surprised by the nuanced ways in which these dimensions played out in practice. Additionally, they were struck by how much some dyads seemed to contradict one another on certain themes, revealing unexpected complexities in the therapeutic relationship.

## RESULTS

We developed four themes and six subthemes that highlight the significance of gender differences in the experiential accounts of female patients and their male therapists. At the same time, some participants—sometimes even the same individuals—expressed that gender played only a secondary role in psychotherapy. As human beings, the participants naturally conveyed complex and occasionally contradictory experiences. From a phenomenological perspective, this illustrates the multifaceted nature of lived experience.

Table 3 presents the Group Experiential Themes along with their corresponding subthemes. For each theme, the participant groups contributing experiential statements are identified. The table also highlights cases where members of the same dyad expressed similar perspectives. Additionally, the number of participants and dyads associated with each theme is shown in brackets, with a maximum of five patients, five therapists and five dyads.

**TABLE 3:** Group experiential themes and subthemes.

Theme	Subthemes	Participant groups
1. Gender shapes the topics and interventions in psychotherapy	1.1. Gender plays a crucial role in the discussion of certain topics 2.1. Therapeutic interventions are shaped by gender	Female patients (4) Male therapists (3) Similar perspective within a dyad (3)
2. The male therapist's gender as valuable for the female patient	2.1 The value of a ‘male’ perspective in exploring female patients' intimate relationship issues 2.2 Addressing difficult patient–father relationships	Female patients (4) Male therapists (3) Similar perspective within a dyad (2)
3. The female patient's gender as valuable for the male therapist	3.1 Learning about contemporary women's experiences 3.2 Working with women is emotionally easier	Male therapists (2)
4. Attraction between male therapist and female patient	—	Male therapists (3)
5. Gender plays a lesser role in psychotherapy	—	Female patients (4) Male therapists (3) Similar perspective within a dyad (2)

The results in the main text are presented using an adapted version of Rodgers and Cooper's (2006) ‘Scoring Scheme for Qualitative Thematic Analysis’, which translates numerical data into verbal descriptions while preserving the complexity of qualitative findings. Verbal descriptors with corresponding numbers are all (*n* = 5), most (*n* = 4), some (*n* = 3), a couple (*n* = 2) and one (*n* = 1).

### Gender shapes the topics and interventions in psychotherapy

Most patients and some therapists agreed that gender influenced the topics and interventions in psychotherapy, with this consensus appearing within some dyads. This was particularly evident in how sensitive and intimate issues were addressed. Therapists also observed that interventions often varied based on the patient's gender.

#### Gender plays a crucial role in the discussion of certain topics

A female patient expressed difficulty discussing sensitive issues with men

I would have preferred to work with a woman. … Men are somehow, I don't know, a bit alien to me. … I trust women more because a woman understands how another woman thinks, right? Men think differently … I'm used to talking about difficult issues only with women.

Men seemed strange to this patient, which is why she preferred discussing sensitive issues with women. However, later in the interview, she acknowledged that after initial difficulties, ‘it was okay now’ with her male therapist. Interestingly, her therapist had a different view of their relationship. Contrary to her statement, he believed that she did not have a problem with men.

Members of another dyad acknowledged that gender differences influenced the discussion of certain topics. The patient, who had previously worked with both male and female therapists, stated, ‘When it comes to really intimate topics, it's certainly easier to talk to a woman’. She attributed this to learned gender roles. Her therapist, in his own interview, agreed, saying, ‘It will certainly have an influence, as one might speak and respond differently to a woman than to a man’. However, both the patient and therapist in this dyad had similar views that gender generally remained in the background of their therapeutic relationship.

Similar to the previous dyad, another patient believed that factors beyond gender were more important in psychotherapy. She emphasized that a strong interpersonal relationship mattered more than gender differences. However, she also expressed reluctance to discuss very intimate issues with her therapist:

I would have preferred not to talk much about sexuality, for example. I believe it's more comfortable to discuss that with a woman. Yes, when it comes to these very intimate issues, I feel more at ease with a woman.

Her therapist not only agreed that gender mattered pertaining to some topics, but rather experienced gender differences more generally, ‘I believe that it always matters whether it's a man or a woman sitting here’.

Another patient mentioned that she generally had no issues discussing topics like sexuality and menstruation with her therapist. However, she acknowledged that how she addressed these topics depended on her interlocutor's gender: ‘With a woman, I would likely speak differently… because a woman can empathize. Maybe she faced the same problem herself’. She also emphasized that these intimate issues were not the focus of her therapy and thus did not pose a problem. Her therapist agreed that gender made a difference for him. When asked whether it was easier or more difficult working with a woman, he compared her to a male patient he had recently seen.

In principle, working with men is somehow easier for me. It feels more direct, unfiltered… I talked with this male patient about relationships, and we could discuss things without inhibition. With this female patient, I have more inhibitions, I must admit. I'm perhaps more careful.

By noting that he felt more restrained discussing intimate relationships with his female patient than with a male patient, he suggested that gender dynamics influenced the therapeutic process.

Gender appeared to influence the discussion of sensitive issues in psychotherapy. Female patients often preferred discussing intimate topics, such as sexuality, with women. One patient shared that she found men unfamiliar and was used to confiding only in women, whereas another noted that she could speak more openly about menstruation with a female therapist due to their shared bodily experiences. Although one dyad described gender's role as subtle, they acknowledged it influenced how certain topics were addressed. These patterns suggest that past experiences in their lived spaces, inscribed into intercorporeal memory, shaped their phenomenal fields. This gendered phenomenal field made patients—and at least one therapist—more comfortable addressing sensitive topics in gender‐homogeneous dyads.

#### Therapeutic interventions are shaped by gender

Participants not only argued that gender differences played a role when it came to sensitive issues; a couple of therapists stressed that gender was also relevant when it came to therapeutic interventions.

When asked if his patient's gender influenced his interventions, a therapist acknowledged a tendency to approach men and women differently:

This might be a bit stereotypical. [With a woman], it often starts with compassion, understanding, and letting her talk. With a man, depending on the individual, the focus may shift more towards action, especially if focusing on emotions triggers too much fear. … Of course, assessing a woman's capacity is more important, then perhaps moving towards action. And with a man, who might be more action‐oriented—though this is very stereotypical—… [I would] ask: “How are you doing now? What's the matter? What do you feel?” to help develop that side.

While the therapist acknowledged that these were gendered stereotypes and added a caveat, he nonetheless identified a gender‐specific tendency in his interventions with some patients.

Shifting focus to therapy materials, another therapist noted differences in his patients' choices when using objects in interventions: ‘In my experience, women value aesthetics. And that was true for her; she tended to choose beautiful, delicate objects’. Additionally, when working with body‐focused interventions, the same therapist observed that male patients were generally more assertive and aggressive, while his female patient appeared more cautious in her approach.

These therapists' statements highlight how interventions can be gendered within the interactive phenomenal field. This includes both the expected gendered responses from patients and the use of interventions by therapists that are supposed to meet gender‐specific needs.

### The male therapist's gender as valuable for the female patient

Most patients—and some therapists—observed that the male therapist's gender could be a therapeutic resource. A couple of dyads, in particular, highlighted two key aspects. First, the therapist's lived male experience, embodied through cultural practices of masculinity, offered a complementary gendered perspective on the female patient's intimate relationship issues. Second, his presence opened a space for exploring challenging father‐related dynamics.

#### The value of a ‘male’ perspective in exploring female patients' intimate relationship issues

While most female patients preferred not to discuss intimate issues like sexuality with their male therapists, many found it helpful to gain a ‘male perspective’ on their relationships during therapy.

A patient, who had previously seen female therapists, chose a male therapist this time for a ‘different perspective’. Her therapist agreed, noting, ‘She asked me a lot about what boys are like, what they think if she behaves or acts in a certain way’. Both participants implied that the therapist's ‘male’ perspective on the female patient's intimate relationships had its merits in therapy.

Another patient emphasized this aspect in a particularly difficult intimate relationship:

I believe it helped that he was also a man… I think his confirmation was important—that it's actually not quite ordinary from a male perspective when someone says, “I love you so much, I must be with you, and I wouldn't know what to do if you left me,” and then it all falls apart.

From this patient's perspective, validation from her male therapist that her former partner's behaviour was unusual proved to be assuring.

Similarly, another patient agreed. When asked if there were topics where talking to a male therapist was easier than with a female therapist, she noted that a male perspective on partnership could be helpful, particularly on the topic of infidelity: ‘When the male partner is cheating, I think I would ask myself why. And I think a man could offer an answer’.

Another patient initially preferred a female therapist, but over time, she believed trust had developed, and her therapist's ‘male’ perspective had become valuable. When asked if it was helpful in any situation that her therapist was a man, she replied

It is helpful concerning relationship and love issues. He [her therapist] told me how he sees me as a woman from a male perspective. … I also believe that some views of a man are perhaps different from those of a woman, right? Men think differently, after all.

Finally, a therapist in another dyad offered a complementary perspective from the therapist's side: ‘We also talked about sexuality… I had the impression that it was helpful that I am a male therapist’. Notably, however, his patient was the only one who did not describe a ‘male’ perspective as helpful.

Participants acknowledged the value of a contrasting gendered perspective, such as a male viewpoint on relationship issues involving men. This suggests that patients and therapists are aware of the differing gender‐specific experiences in the lived space, which shape distinct phenomenal fields that offer fresh perspectives on patients' relationship concerns. This contrasts with the initially expressed preference for gender–homogeneous dyads when addressing intimate topics like sexuality. Depending on the context, patients and therapists valued either gender‐specific similarity or difference in their interactive phenomenal fields.

#### Addressing difficult female patient–father relationships

Some interviews hinted that a difficult relationship with the patient's father emerged as a topic that could be beneficially explored with a male therapist though the issue remains ambiguous.

A patient expressed conflicted feelings towards her therapist:

My relationship with my father is not good, and that's something we often discuss. And that's helpful. However, I know he has children, and sometimes I think, “[he is] a man who has children, he is a good father.” That thought lingers with me. … It's such a strange feeling, and I'm not sure if it's jealousy, envy, or longing.

While she found it helpful to discuss her troubled relationship with her father, she felt that an attentive father was missing in her own life by seeing her therapist as ‘a good father’. Her therapist, in his own interview, referred to the same issue, noting, ‘She sees … me as a man who pays attention to her, who doesn't disregard or reject her. There are many layers to this. It's possibly a father transference’.

In another dyad, the patient shared that she initially did not want to work with a male therapist because ‘I just had a distanced relationship with my father’. Her therapist acknowledged this difficult relationship and addressed it with her in therapy.

Participants' statements align with the notion that past experiences with significant others, inscribed in the body memory, shape the challenges and opportunities in the interactive phenomenal field of therapy. In one dyad, the patient's experiences with her father seemed particularly significant. These experiences, actualized in the phenomenal field, triggered challenging emotions but also created an opportunity to address parent–child relationship issues and may provide the patient with a corrective emotional experience.

### The female patient's gender as valuable for the male psychotherapist

Complementing the previous theme, this theme explores how male therapists might perceive the female gender of their patients as uniquely valuable. A couple of therapists expressed such perspectives. On one hand, female patients provide male therapists with opportunities to better understand and learn about women's lived experiences. On the other hand, women might be perceived as more emotionally accessible in psychotherapy.

#### Learning about contemporary women's experiences

A therapist highlighted how working with a female patient allowed him to gain new insights into contemporary women's experiences:

I wasn't familiar with the online dating she mentioned. It was like, “Okay, I really need to understand this better.” … I learned a lot about … how she develops friendships and communicates with women and female colleagues, what they discuss … what it means to be a young woman, and how she navigates intimate relationships as a woman. … These insights into the world of women are new to me.

The patient recognized the therapist's unfamiliarity with online dating:

The first time I talked about boys, it felt a bit funny—maybe because of the age difference or generational gap. When I mentioned meeting my current boyfriend online, my therapist referred to it as “a relationship” after just a week. I had to explain, “No, it's different today.”

This dyad's experiences highlight generational and otherwise experiential gaps between male therapists and female patients, creating opportunities for learning. The therapist's openness to exploring his patient's lived space, particularly in unfamiliar areas, provided valuable insights into the evolving dynamics of young women's lives. This expanded the therapist's interactive phenomenal field by enabling new ways of perceiving and relating to younger female patients.

#### Working with women is emotionally easier

A therapist found it emotionally easier to work with women:

Perhaps it is emotionally easier to engage [when working with female patients]. … It could be because there's less competition. … With men, it's sometimes more difficult for me to stay with an issue, to sense, touch, and hold onto it because the connection tends to fade faster. And perhaps I allow myself to be pulled away more easily. It might be an inner readiness to sense more. Maybe it's related to my own experiences with my mother, where things were more tactile, physical, and emotionally charged.

This therapist perceived less competitive dynamics with women than with men in therapy. He noted a deeper and more stable emotional connection with women, potentially influenced by his own experiences with his mother. Phenomenologically, his intercorporeal memories from these childhood interactions may have shaped his phenomenal field, allowing for greater emotional resonance with women in therapy.

### Attraction between male therapist and female patient

Some therapists reported feeling a degree of attraction towards their female patients. However, none of the patients expressed experiencing similar feelings of attraction towards their psychotherapist during their interviews.

When asked about the influence of gender on their therapeutic interventions, one therapist acknowledged occasional instances of flirtation: ‘Perhaps sometimes a smile or a bit of flirting happens’. In contrast, his patient described the interaction as more detached, stating, ‘He acted very professionally and neutrally’. This contrast highlights a divergence in the interactive phenomenal field: while the therapist perceived flirtation, the patient did not report any flirtatious behaviour, suggesting maintained professional boundaries.

Another therapist recalled, ‘There was a certain sexual attraction, but I couldn't pinpoint the reason until I discussed it with him [his supervisor]. It turned out to be a kind of attachment’. While the therapist initially experienced sexual attraction, he reframed it with his supervisor's support as a form of emotional bond.

Similarly, a therapist of another dyad reported, ‘The positive atmosphere in the therapeutic relationship could easily be facilitated by this man‐woman dynamic. … These are, as psychoanalysts say, transference processes that likely emerge with sufficient sympathy. Analysts refer to this as transference love’. This therapist employed psychodynamic terminology to describe the phenomenon he observed between himself and his patient.

Notably, none of the female patients reported experiencing attraction toward their therapists. This absence may reflect either a lack of attunement to an underlying atmosphere of attraction within the interactive phenomenal field or an active avoidance in acknowledging such feelings during the interviews. Female patients might have internalized cultural norms that discourage discussing attraction in professional contexts. Moreover, while male therapists might interpret certain gestures as indicative of attraction, female patients may simply perceive them as expressions of kindness. All of these responses are likely underpinned by distinct gendered intercorporeal memories. In contrast, therapists, shaped by their training, may feel less constrained to engage with attraction more openly within their phenomenal field.

### Gender plays a lesser role in psychotherapy

Most patients and some therapists noted that, in certain contexts, gender played a minor role in their psychotherapy. This contrasting theme highlights the participants' complex and sometimes conflicting experiences. This view was shared within a couple of dyads. Participants viewed gender as less influential in the therapeutic relationship, emphasizing the greater importance of personal and relational factors.

A dyad agreed that factors other than gender differences were more important in their therapy. The female patient explained

I'm unsure if a difference between men and women exists in therapy… Human beings need to match, and trust must be there. While there are differences between men and women, they think differently, in this case, gender didn't matter for my ideas or my goals.

Although she acknowledged general gender differences, the patient emphasized that trust, personal compatibility and her therapy goals were more significant. Similarly, her therapist believed that the patient's ‘biographical background played a role’ and was more important than gender in their therapeutic process.

Similarly, another patient agreed that her personal biography and family issues were more important than gender. When asked about the influence of her therapist's gender on her therapy, she responded: ‘I can talk with him about everything. I don't feel restricted from discussing things like menstruation or sexual topics’. Her therapist observed her behaviour in a similar light: ‘She talks quite openly about contraception and such things, actually very openly. This is almost unusual for me in this process’. While both members of the dyad described their experiences in parallel, the therapist described his patient's openness as ‘unusual’. This indicates a dissonance in the interactive phenomenal field regarding the patient's openness. Despite the patient's view that gender was not significant, her therapist implicitly held a different perspective. This was evident not only in his observation that it was unusual for a female patient to openly discuss female bodily processes, but also in his preference for working with male patients, whom he perceived as having a greater capacity for direct communication.

Another dyad agreed that gender differences were relatively inconspicuous in their therapeutic relationship. When asked how the female patient experienced working with her male therapist and whether gender played a role, she replied, ‘I don't think there's a difference’. Her therapist expressed a similar view: ‘For me, other individual factors are more in the foreground … than whether a man or a woman is sitting in front of me’.

In a similar vein, another therapist saw gender differences as less important, though he acknowledged that they might matter to some of his patients:

For me, it makes no difference— a human being is a human being. … Whether a woman, a man, or whatever. … If a woman has issues with men and seeks my help, I address it… I didn't get the impression that she [his patient] had an issue with men.

However, his patient held a different view on the importance of gender in therapy. She found men somewhat unrelatable and would have preferred to work with a female therapist.

Another patient emphasized that she tried to view ‘male and female therapists as a unit and not so much in terms of gender’. However, her therapist held a different perspective, noting that it always mattered to him whether he was working with a male or female patient.

These diverse perspectives highlight that while several participants viewed gender as a background factor or less significant than other aspects of therapy, others recognized its considerable influence. Interestingly, some participants expressed both seemingly contradictory views within the same interview. This suggests that past gendered experiences of both patients and therapists may shape the therapeutic process to varying degrees and reflect differing levels of awareness about how gender influences an individual's interactive phenomenal field. How this, in turn, affects the therapeutic process or relationship remains open to discussion.

## DISCUSSION

Several participants acknowledged that gender plays a meaningful role in shaping both the topics explored and the therapeutic interventions applied. For example, female patients found it more challenging to address intimate issues with male therapists, and at least one male therapist reported feeling more restrained when discussing intimate relationships with female patients. These participants' past gendered experiences in their lived spaces appeared to have influenced the comfort levels and dynamics within gender–heterogeneous dyads. This observation aligns with previous research. Studies have found that therapists often face challenges when addressing intimate issues in mixed‐gender pairings (Schigl, 2019; Tolle & Stratkötter, 1998). Viewed through the lens of the phenomenal field, it appears that women may lack a history of resonant, supportive interactions with men regarding intimate topics, rendering such discussions alien. In contrast, female patients have reported feeling more at ease with female therapists, who share similar social and embodied experiences (Schweitzer et al., 2024). The present study similarly indicates that the absence of shared embodied experiences between female patients and male therapists may contribute to these dynamics. Overall, these findings help explain why previous research has observed lower satisfaction among female patients in gender‐heterogeneous pairings (Johnson & Caldwell, 2011; Leitner et al., 2013; Schigl, 2014) and a preference among female patients for female therapists (Ilagan & Heatherington, 2021; Landes et al., 2013).

Regarding intervention styles, male therapists noted that their approaches varied depending on the patient's gender; for instance, by using a more supportive approach with female patients. This suggests an anticipation of a gendered patterning in the phenomenal field of patients, allowing therapists to respond more effectively when interventions were tailored to their patients' gender. This observation aligns with other research highlighting the gendered nature of interventions and their differential effects on patients depending on their gender identity (Gehart & Lyle, 2001; Ogrodniczuk & Staats, 2002; von Wyl et al., 2021).

Furthermore, participants indicated that the male therapist's gender could be an asset in therapy. They reported that his perspective provided a complementary lens for addressing the intimate relationship challenges faced by female patients. Implicitly, this suggests that the distinct gendered experiences of patients and therapists—each shaped by their unique lived spaces—contribute to the formation of differently configured phenomenal fields. The presence of contrasting perspectives appeared to facilitate fresh insights into patients' relational issues. Depending on the context, participants valued either shared or divergent experiences in their phenomenal fields—a nuanced finding that has not been previously addressed in existing research.

Additionally, the male therapist may be perceived as ‘good father’ by their female patients, providing opportunities to explore unresolved issues related to the patient's father or other male significant others. This finding corresponds with prior research in which male therapists aimed to serve as positive male role models for female patients (Schigl, 2019). Such dynamics offer the potential for therapeutic reparenting but also pose the risk of idealization. The question remains how much therapists contribute to being perceived as the better father or partner, with some studies suggesting that male therapists may be inclined to adopt this role (Schigl, 2018).

Complementing the benefits associated with a male therapist's gender, a couple of therapists also recognized the therapeutic value of working with female patients. For a male therapist, engaging with a female patient offered a unique opportunity to delve into women's lived experiences—a perspective rooted in their distinct gendered lived space. These experiential differences, shaped by diverse lived realities, foster opportunities for mutual learning and the expansion of the therapist's phenomenal field. In doing so, therapists may gain fresh insights and develop new ways of perceiving and relating to other female patients. While this observation appears novel in the literature, from a phenomenological standpoint, encounters with differing lived spaces, regardless of gender, have the potential to broaden a therapist's perspective.

Another therapist noted that he found his female patients to be more emotionally accessible and easier to engage with. Schigl (2019) reports that male therapists often find it easier to establish strong therapeutic relationships with female patients compared to male patients. Similarly, psychotherapists have observed that male patients frequently appear withdrawn and discouraged, particularly during the early stages of treatment (Seidler et al., 2021).

Therapists acknowledged a distinct relational dynamic in male therapist–female patient dyads, noting varying degrees of sexual attraction towards their female patients. This phenomenon is frequently observed in such therapeutic relationships, as highlighted in previous research (Pope et al., 2006; Schigl, 2019). Notably, none of the interviewed patients reported experiencing similar feelings of attraction. This contrast highlights a divergence in the interactive phenomenal field—specifically, how male therapists and female patients perceive and experience each other during therapy sessions. This tendency could reflect subtle manifestations of hegemonic masculinity within the male therapist–female patient dyad (Proctor, 2008). If left unexamined, misinterpretations may lead to iatrogenic effects, as reported by Leitner et al. (2013). However, the therapists in the present study appeared to engage in reflective practices to contextualize and manage feelings of attraction within their therapeutic frameworks. These differing perceptions underscore the ongoing need for consistent, open feedback throughout the psychotherapeutic process (Claiborn & Goodyear, 2005). Repeatedly checking in helps therapists better understand how their interventions are received and provides patients with the opportunity to clarify their own experiences within the therapeutic relationship.

Finally, several participants noted that gender sometimes played a minor role in psychotherapy, whereas others highlighted its significant impact—occasionally expressing both views within the same interview. Participants' perception of gender as a minimal influence on therapy may help explain why several studies have found no significant ‘gender effect’ on therapy outcomes (Blow et al., 2008; Lambert, 2016; von Wyl et al., 2021; Zlotnick et al., 1998). In the present study, several participants described gender as a subtle, background factor within the therapeutic relationship, while other elements—such as age differences or specific biographical backgrounds—may have carried greater significance. These differing perspectives reflect a spectrum of lived experiences regarding gender. At times, gender appears to recede into the background of the phenomenal field, becoming less salient in the therapeutic process. Yet, with shifts in attention or context, gendered dynamics may once again emerge as experientially present, while other relational dynamics—rooted in participants' unique body memories—come to the fore. These individual and dyadic variations highlight how interactive phenomenal fields are shaped by patients' and therapists' past gendered experiences in their respective lived spaces. A mismatch in the perceived relevance of gender between patient and therapist could create barriers to open communication and foster feelings of alienation (Tolle & Stratkötter, 1998). However, when these differences are recognized and explored, they may also open new possibilities for understanding and growth, enriching the interactive phenomenal field. Participants in this study expressed diverse—and at times conflicting—perspectives on the role of gender in outpatient psychotherapy. While many acknowledged its relevance, some emphasized other factors as more central to their therapeutic experience. The transferability of these findings should be considered in light of the study's modest sample size.

### Limitations and implications for research

The patients were in the initial phase of psychotherapy, a period during which gender may play a more prominent role (Schigl, 2018). This may have amplified the perceived significance of gender in the context of this study. Moreover, previous therapy experiences and biographical encounters with men were not explicitly addressed in the interviews. However, past negative experiences or trauma involving men may have shaped patients' current experiences within the therapeutic dyads. While the sample size may appear small from the standpoint of quantitative or mixed methods research, it is appropriate for the chosen phenomenological framework. Nevertheless, a larger and more diverse sample—particularly with respect to gender identity and educational background—could enrich the scope and applicability of the findings. The absence of openly LGBTIQA* participants and those with less formal education limits the exploration of how these insights apply in different contexts. Including such perspectives in future research could reveal important nuances. Additionally, the lack of therapists with behavioural and systemic orientations leaves potential differences between therapeutic approaches unexplored. Examining these distinctions could provide a broader understanding of therapeutic dynamics.

### Implications for practice

Two findings from the present study may hold particular relevance for psychotherapy practice, as they have not been extensively explored in prior research. First, therapists might engage in therapeutic work with patients of another gender identity to intentionally gain deeper insight into another gender group's lived experience. Such interactions can broaden therapists' relational capacities within the therapeutic context, fostering greater attunement to diverse perspectives. This approach need not be limited to gender; it can also extend to other experiential backgrounds, such as generational or cultural contexts. Second, therapists may offer a gendered perspective on the relationship challenges faced by patients of a different gender identity. However, it is crucial they clarify that this is *a* perspective, not *the* perspective of ‘a gender’. Therapists should remain mindful that their views are shaped by cultural practices of masculinity and femininity within their lived space (McNay, 2004).

A therapist's openness to understanding the lived experiences of patients of another gender identity and, when invited, offering a gendered perspective can enrich therapy—if done with consent and reflexivity (Tolle & Stratkötter, 1998). In this study, participants either downplayed or highlighted gender differences without exploring how these dynamics could be addressed or reshaped in therapy. Instead of viewing gender differences as fixed or irrelevant, they could be seen as fluid and adaptable. Psychotherapy training could support this shift by placing greater emphasis on gender issues, a topic currently underrepresented among practicing psychotherapists in Austria (Schigl & Ahlers, 2023).

## AUTHOR CONTRIBUTIONS

**Erwin Schweitzer:** Formal analysis; investigation; methodology; writing – original draft; writing – review and editing. **Yvonne Schaffler:** Conceptualization; writing – review and editing; formal analysis; data curation; investigation; methodology; project administration. **Thomas Probst:** Conceptualization; methodology; writing – review and editing. **Elke Humer:** Conceptualization; methodology; writing – review and editing. **Christoph Pieh:** Writing – review and editing. **Brigitte Schigl:** Conceptualization; methodology; supervision; writing – review and editing.

## FUNDING INFORMATION

This research received no external funding.

## CONFLICT OF INTEREST STATEMENT

The authors have no conflicts of interest to disclose.

## ETHICAL APPROVAL

This study was approved by the Ethics Committee of the University of Continuing Education Krems (UWK), Austria (EK GZ 28/2018‐2021).

## INFORMED CONSENT FROM PARTICIPANTS

Participants provided written informed consent.

## Data Availability

The full transcripts of the qualitative interviews conducted in this study are not publicly available to protect participant anonymity and confidentiality. However, anonymized excerpts or summary data may be provided upon reasonable request to the corresponding author, subject to ethical and confidentiality considerations.
